# Migration tactics affect spawning frequency in an iteroparous salmonid (*Salvelinus malma*) from the Arctic

**DOI:** 10.1371/journal.pone.0210202

**Published:** 2018-12-31

**Authors:** Colin P. Gallagher, Kimberly L. Howland, Stephen J. Sandstrom, Norman M. Halden

**Affiliations:** 1 Fisheries and Oceans Canada, Winnipeg, Manitoba, Canada; 2 Ontario Ministry of Natural Resources and Forestry, Bracebridge, Ontario, Canada; 3 Department of Geological Sciences, University of Manitoba, Winnipeg, Manitoba, Canada; Shanghai Ocean University, CHINA

## Abstract

Otolith strontium and multi-year mark-recapture information were used to characterize associations between migration patterns and spawning frequencies in an anadromous Dolly Varden (*Salvelinus malma*) population (Rat River, Northwest Territories, Canada) that undertakes a long migration between freshwater spawning/overwintering (Fish Creek; a tributary to Rat River) and marine feeding habitats (Beaufort Sea) (~800 km round trip). Reconstructions of lifetime annual migration histories among otolith annuli was matched to information on reproductive status (current-year ‘spawner’ or ‘non-spawner’) that was known in two different, sometimes successive, years for each fish. Two migratory life histories were observed: fish either migrated annually after smoltification or periodically skipped an annual ocean migration to remain in freshwater and spawn. Different spawning frequencies were detected where fish not migrating annually tended to spawn in alternate years (84.6%) more often than those migrating annually (50%). Additionally, annually migrating fish had lower longevity (≤9 years vs. ≤13 years). The evaluation of differences in spawning frequency between sexes, independent of migration tactic, revealed males (84.6%) skipped spawning more often than females (51.2%) suggesting fitness trade-offs between life histories differ between sexes. Further, some fish returned from the sea considerably earlier than the majority of other current-year migrants. Our findings demonstrate intrapopulation diversity in migration behaviour and reproductive frequency.

## Introduction

Migratory tactics in animals produce life history trade-offs in patterns of resource allocation towards maintenance, growth, and reproduction that affect morphological, physiological, and behavioral traits to maximize fitness [1–4]. Tactics vary extensively among and within species and populations [5,6]. One example of diversity within populations is migration dimorphism (‘partial migration’) where a population is composed of a mixture of resident and migratory individuals that can lead to differences in body size between migratory phenotypes (e.g., smaller-sized resident and larger-sized migratory phenotypes in salmonids) [7].

In fishes, migration can provide advantages in both semelparous and iteroparous species such as increased size and growth, fecundity, longevity, energy stores, and access to alternative suitable habitats. However, one of the most important costs of migration is increased probability of mortality (e.g., [8,9]). Characterizing trade-offs in migration, particularly trade-offs that affect reproduction, has implications for population dynamics and life history theory that are relevant for the management and conservation of migratory species.

In fishes, greater migration distance and or difficulty result in trade-offs in reproductive investment including reduced male and female gonadal investment, smaller egg size, reduced investment in secondary sexual characters that affect male breeding success, and increased incidence of alternate-year spawning (i.e., skip spawning) ([9–11], and references therein). Alternate-year spawning has been documented in many iteroparous teleost fishes and is mainly a result of insufficient energy accumulation and suboptimal environmental conditions [12]. Benefits of alternate-year spawning may include higher survival and opportunity for growth, and increased lifetime reproductive output in females and therefore higher overall fitness [12].

Energy accumulation in Arctic aquatic ecosystems is expected to be relatively challenging for costly functions such as migration and reproduction for anadromous iteroparous fishes from the sub-family Salmoninae as evidenced by the positive correlation between frequency of alternate-year spawning and latitude in brown trout (*Salmo trutta*) [13]. Iteroparous salmonines in the Arctic are known to exhibit repeat (i.e., consecutive years) and or alternate-year spawning within a population ([14,15] and references therein). For example, some anadromous Arctic charr (*Salvelinus alpinus*) are known to skip annual ocean migrations for the purpose of spawning [15,16]. Specifically, individuals will forgo a feeding migration to the sea (i.e., skip migration) and remain in freshwater to spawn but will resume migration the following year yet be unable to repeat spawning (i.e., will spawn in alternate years) due to the high energetic costs associated with spawning and overwintering [17]. Similarly, skipping migration for the purpose of spawning has also been documented in northern Dolly Varden (*S*. *malma malma*) but only in anadromous populations inhabiting river systems in Alaska [18–20]. Alternatively, the majority of Arctic charr from the Island of Spitsbergen (Norway) migrate annually after smoltification (first time to sea) [16].

Otolith chemistry, specifically strontium (Sr) concentration or the ratio between Sr and calcium (Ca), has been useful in characterizing lifetime diadromous migration history, and has revealed complex migration patterns and habitat utilization in multiple species (e.g., [21–23]). However, there has been little research examining migration and spawning frequency (e.g., skip migration/alternate-year spawning) in anadromous salmonids. Specifically, research using methods (e.g., Sr in otoliths and mark-recapture) where annual migration patterns and reproductive status of individual fish are simultaneously known over multiple years enables the evaluation of reproductive life history consequences of different migration tactics [24].

**Fig 1:**
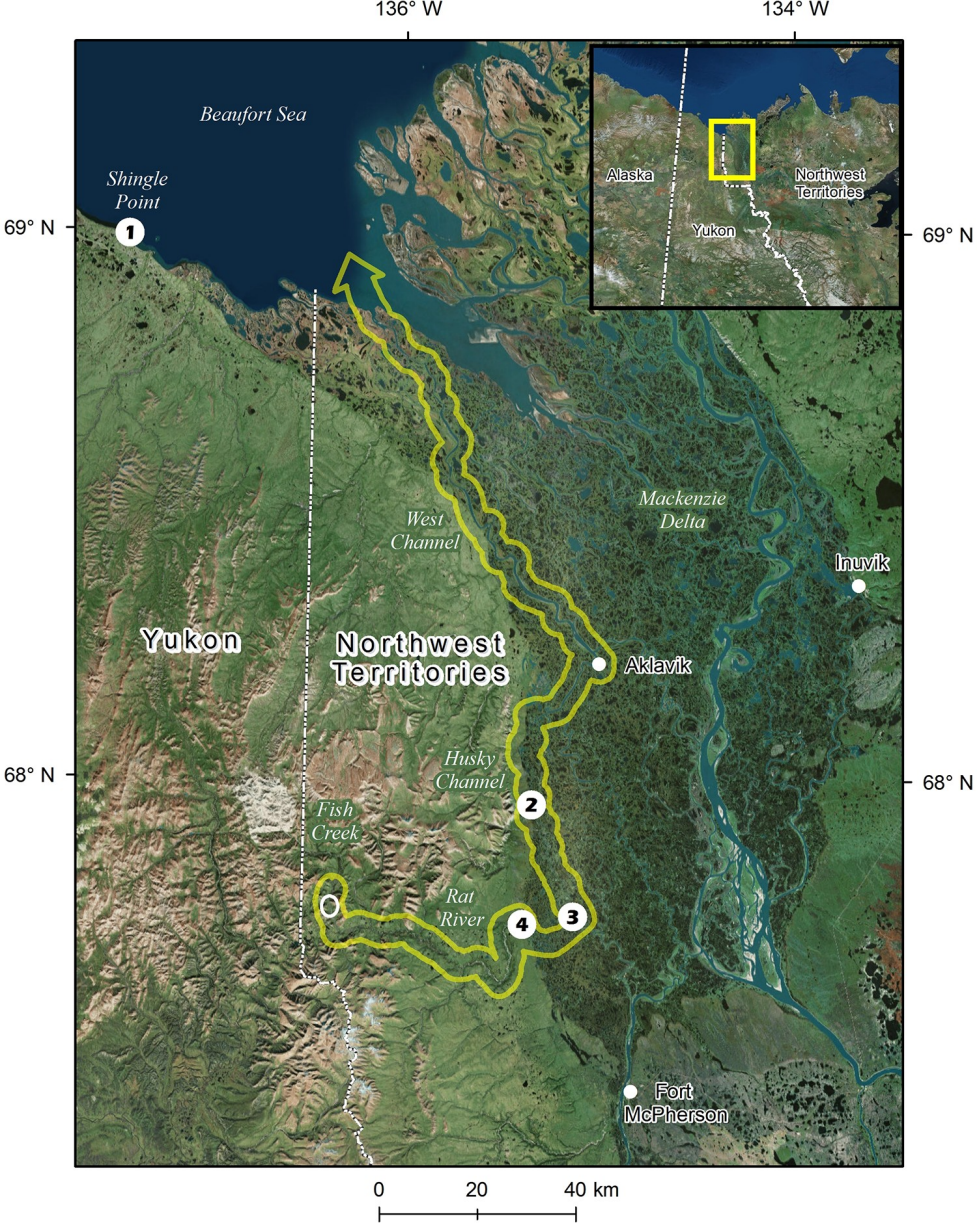
Map of study area showing freshwater migration corridor (yellow outline arrow; pointing towards direction of ocean migration) between spawning and overwintering location (open white circle) in Fish Creek and feeding areas in the Beaufort Sea utilized by anadromous Dolly Varden from the Rat River stock. Harvest monitoring locations (numbered closed circles) at (1) Shingle Point (Beaufort Sea), Mackenzie Delta (Husky Channel) at (2) Big Eddy, and in the Rat River at (3) the mouth and (4) Destruction City. Map created using ArcGIS 10.4.1 for Desktop; service layer credit: Esri, HERE, DeLorme,Mapmyndia; source: Esri, DigitalGlobe, GeoEye, Earthstar Geographics, CNEAS/Airbus DS, USDA, AeroGRID, IGN, and the GIS User Community (images taken on 4 August 2010, 28 May 2011, 17 July 2014, and 23 August 2015).

Anadromous northern Dolly Varden from the Rat River, a population situated in the western Canadian Arctic, undertakes annual seasonal migrations between alpine freshwater spawning/overwintering and marine habitats upon smoltification. Spawning occurs in Fish Creek, a tributary to the Rat River, during fall. After the overwintering period, fish will migrate in spring down the Rat River and Mackenzie Delta to feed in the Beaufort Sea and will typically begin their return migration to Fish Creek later in the summer (Fig 1). Dolly Varden are harvested in subsistence fisheries by both Inuvialuit and Gwich’in people during summer in the Beaufort Sea and over the duration of the return migration in the Mackenzie Delta and Rat River. The Rat River population undertakes among the longest freshwater migrations for northern Dolly Varden in North America. It is unknown how the migration distance influences the migration and reproductive characteristics of the population. Unlike Dolly Varden in Arctic Alaska [18–20], little is known about annual seaward migrations in populations further east and almost nothing is known about how migration patterns affect spawning frequency in this species. If Dolly Varden from the Rat River exhibit variation in annual ocean migration patterns we predict differences in spawning frequencies and longevity. Specifically, a high degree of alternate-year spawning would be expected if energetic cost and mortality associated with migration and reproduction were high [9]. Higher levels of mortality would be expected for fish that migrate more often in consecutive years than those who periodically forgo ocean migration [8]. In this study we characterized annual migration patterns and associated spawning frequency of anadromous Dolly Varden from the Rat River (henceforth abbreviated to RR) by overlaying data from Sr patterns among otolith annuli and reproductive status (current-year ‘spawner’ or ‘non-spawner’) known in two different, sometimes successive, years obtained through a multi-year mark-recapture study. This unique data set allowed us to evaluate whether fish from the RR population periodically forgo ocean migration prior to a spawning event or migrate annually after smoltification, and if spawning frequency differed between these two migration tactics. We also tested whether migration patterns were associated with differences in age-at-first migration and maternal pre-spawning habitat to elucidate possible reasons for these patterns, and differences in longevity to ascertain additional trade-offs due to anadromous migration strategies. Furthermore, we evaluated differences in spawning frequency between sexes to determine possible consequences of migration tactics in females and males. Our findings will add new insights into bet hedging strategies, and the trade-offs between migration and reproduction and its impact on longevity.

## Methods

### Species background

In Canada, populations of anadromous northern Dolly Varden are genetically differentiated among river systems and highly philopatric (i.e., tendency to return to river of origin after ocean feeding migration) [25]. These populations exhibit partial migration where an anadromous (female biased) and stream resident (almost exclusively males, typically <310 mm fork length (FL) and spends entire life in freshwater) phenotype spawn and overwinter in sympatry. Spawning and overwintering habitat are inextricably linked and occur in alpine headwater reaches in areas associated with perennial ground water springs that maintain unfrozen stretches of river throughout the winter [26]. After rearing in natal streams for 2–5 years, individuals undertake migrations to feed in the sea each spring (usually late June and July) during ice breakup and typically return to freshwater habitats from mid-summer (middle of July) to late-fall (middle of September) to spawn and or overwinter [14,20].

Male and female life history characteristics of the RR population include a maximum size of approximately 767 mm and 720 mm, respectively, length-at-maturity of approximately 510 mm and 495 mm, respectively, a similar age-at-maturity of 6 years, a greater longevity and lower annual total mortality (A) in females (maximum age = 13 years, A = 0.47) compared to males (maximum age = 11 years, A = 0.59) based on age frequency data (S1 Fig), and a significantly higher growth rate in males [27].

### Study area and migration distance

Dolly Varden from the RR stock not only utilize Fish Creek (67°45.8’ N, 136°18.1’ W) (Fig 1) for spawning and overwintering but also for the rearing of juveniles [28]. Dolly Varden spawn in proximity to and overwinter under an aufeis field (i.e., mass of layered ice; [29]) situated near the confluence of Fish Creek and RR [28,30]. Apart from Fish Creek, no other streams with groundwater springs providing spawning/overwintering habitat to Dolly Varden have been documented in the RR watershed [26,30]. Additionally, spawning anadromous individuals have been captured in Fish Creek in the fall ~28 river km above the aufeis field west of the Yukon Territory/Northwest Territories border (C. Gallagher, personal observation). In the spring (May-June), anadromous fish travel down the RR and Mackenzie Delta (via Husky and West channels) (~317 river km) to the Beaufort Sea [31], a round trip of up to ~690 river km (Fig 1). Dolly Varden of RR origins have been confidently detected in coastal subsistence fisheries as far west as ~64 km from the Mackenzie Delta (mouth of West Channel) at Shingle Point, Yukon (assuming fish followed the coastline, i.e., coastal km; [32]) [27] (Fig 1), although further distances are conceivable given the possible dispersal into offshore habitat [33]. Upon the return migration (typically end of July to mid-September), Dolly Varden are captured in subsistence fisheries in the Mackenzie Delta at the hamlet of Aklavik, along Husky Channel, near the mouth of the RR, and in the lower reach of the RR (Fig 1). In total, Dolly Varden from the RR stock could undertake annual migrations of just over ~800 km (combined river km and coastal km). No other salmonine species are currently known to inhabit Fish Creek or the upper reaches of the RR. It is noted that Pacific salmon (*Oncorhynchu*s spp.) have only been infrequently reported in the lower RR (i.e., site #3 in Fig 1) ([34] and C. Gallagher unpublished).

### Sampling design

The sampling procedures were reviewed and approved by Fisheries and Oceans Canada Animal Care Committee (Freshwater Institute) that follows the standards established the Canadian Council of Animal Care. Permission to conduct scientific sampling was granted by Fisheries and Oceans Canada with full support from the Gwich’in Renewable Resources Board and Fisheries Joint Management Committee. Anadromous Dolly Varden were captured, tagged, and released over multiple years during the spawning season in the fall in freshwater spawning and overwintering habitat via fisheries independent sampling at one end of the freshwater migration corridor (i.e., white circle in Fish Creek Fig 1). Reproductive condition and sex, if in spawning conditions (see details below), was recorded for each tagged individual. Tagged fish were recaptured in subsistence fisheries in subsequent years in seasons and locations of the migration corridor when these fish were returning from an ocean migration at the time of recapture. Upon recapture, tagged fish were dead-sampled, where a fish was killed with blunt force trauma followed by exsanguination (i.e., incision resulting in sufficient blood loss to cause death), and biological data collected, which included the retrieval of otoliths (for ageing and Sr analysis) and recording sex and current-year reproductive status. Therefore, reproductive status was known in two different, sometime successive, years for individual fish. Dolly Varden (tagged and untagged) were also captured at the spawning/overwintering area and a location in the freshwater migration corridor not previously known to be occupied by anadromous individuals during a period of the open water season when Dolly Varden would presumably be feeding in the Beaufort Sea (mid-July in Fish Creek (open white circle) and late June in Aklavik Fig 1). These fish were also dead-sampled (or subsampled) to collect otoliths and record sex and current-year reproductive status (whenever possible).

Determining ages/years when ocean migration occurred using otolith Sr analysis and overlaying years when reproductive status was recorded using samples with known and unknown ocean migration history in year of capture/recapture allowed us to examine the relationship between ocean migration and reproductive tactics to address study objectives, and help explain why Dolly Varden were encountered in Fish Creek in mid-July and in Aklavik in late June. Furthermore, spawning frequency data obtained by dead-sampling was supplemented by live-sampling (i.e., biological data collected from fish prior to release) of previously tagged females and males that were recaptured during the fisheries independent sampling in the fall in Fish Creek to evaluate differences in spawning frequency between sexes.

### Capture and marking

Anadromous Dolly Varden were captured by seine net in Fish Creek at the end of September or early October immediately after the spawning season annually between 2007 and 2016 (S1 Table). Every year, seining to t-bar tag (a short plastic filament with a small t-shaped end that is typically attached at the base of fins in mark-recapture studies of fishes) and recapture fish was consistently conducted over a five km stretch of the creek (~2.5 km above and below the latitude and longitude in ‘Study Area’) where fish congregated in shallow pools (average maximum depth ~1–1.5 m) (see [30]). We assumed the majority of the entire anadromous population was congregated in the area that was seined given the limited geographic distribution of spawning and overwintering habitat for the stock [35] and because very few or no anadromous Dolly Varden were observed below or above the area in late September or early October. One exception was a small stretch of the creek situated ~28 river km upstream (west of the Yukon Territory/Northwest Territories border) that was inhabited by a considerably smaller number of anadromous Dolly Varden during this time of the year (C. Gallagher personal observation). In 2007, 2009, 2010, and 2013–2016 between 426 and 499 fish (≥300 mm) were annually tagged with individually numbered t-bar anchor tags (Hallprint, Australia) (tag size used on all fish: 20 mm exposed filament length and 28 mm marker length) inserted at the base of the dorsal fin (no use of anesthesia). Fork length and reproductive status (‘non-spawner’ or ‘spawner’) as well as sex if in spawning condition were recorded before release. Differentiating male and female spawners was achieved by visually assessing for distinct colouration (female: dark olive green dorsal surface and pink-purple lateral and ventral surface; male: black dorsal surface and orange-red lateral and ventral surface) and pronounced secondary sexual characters in males (e.g., lateral compression of body, presence of kype, and enlarged teeth). ‘Non-spawners’ were either juveniles or resting adults (i.e., spawned in previous years but will not in the current year) (sex unknown at the time of tagging) and retained their silver marine colouration. The distinct colouration among male and female spawners, and non-spawners yielded very high confidence in the visual categorization of sex and or reproductive status (see S2 Fig,[27] for images).

### Collection of tagged samples with known migration history in year of recapture

Tagged Dolly Varden from the RR were recaptured in subsistence fisheries using gill nets while feeding along the Beaufort Sea coast at Shingle Point (station #1 in Fig 1) (2011–2017) during summer or during their return (upstream) migration in the Mackenzie Delta (station #2 in Fig 1) or RR (stations #3 and 4 in Fig 1) (2008–2017) in late summer and early fall (Table 1) (S1 Table). All tagged fish reported at Shingle Point, Mackenzie Delta, and RR were harvested and dead-sampled although a small number of these were released among years. All fish recaptured at these locations, which are situated far from the spawning area, would have be returning from an ocean migration that had been undertaken in the spring. Biological data (including sex and maturity, and otoliths whenever possible) were collected from harvested fish through monitoring programs for fisheries at Shingle Point, Mackenzie Delta, and RR [36,37] (Fig 1). The timing (starting date was ± ~1 week in some years) and duration (~1 month; Shingle Point = July to early August; Mackenzie Delta and RR = end of July to early September) of monitoring programs were consistent among years and allowed for the random encounter of tagged fish either during marine feeding (Shingle Point) or upstream migration (Mackenzie River and RR) periods. The total number of Dolly Varden from the Rat River harvested for subsistence is relatively low. The most recent population assessment estimated that <400 were harvested (using combination of reported harvests in Mackenzie Delta and Rat River, and genetic-mixed stock analyses of samples collected from Beaufort Sea coastal fisheries) between 2009 and 2014 [27].

**Table 1:** Sample characteristics of anadromous Dolly Varden from the Rat River (majority were tagged individuals from a mark-recapture study) examined to infer 1) the relationship between annual migration patterns and spawning frequency using otolith strontium (Sr) analysis, and 2) spawning frequency between males and females.

Topic	Migration status	Source/location	Location along migration route	Reproductive status at time of dead-sample	Date	Tagging status	Sampling type	Sample size
Migration life history & spawning frequency	Known to have undertaken ocean migration in year of dead-sample	Harvest monitoring programs (Shingle Point, Husky Chan-nel, Rat River)	Marine feeding area & return migration corridor	Current-year non-spawner & spawner^#^	Mid-late July; August; early Sept-ember (2008–2017)	Previously tagged	Dead	112 ^a^
	Unknown whether to have undertaken ocean migration in year of dead-sample (found along migrat-ion corridor at a time when assumed to be feeding at sea)	Fish Creek (harvest independent samp-ling)	Spawning & overwintering area	Current-year spawner	18 July 2011	Untagged & previously tagged	Dead	8
		Aklavik* (indepen-dently submitted by harvesters)	Migration corridor	Current-year spawner & unknown	24 June 2013; 21 June 2014	Untagged	Dead	2
	Total examined for otolith Sr concentration							122
				Reproductive status known in two separate years		Previously tagged	Dead	82^b^
				Reproductive status known in two successive years		Previously tagged	Dead	40^c^
Spawning frequency between sexes	Known & unknown	Harvest monitoring programs & Fish Creek (harvest dependent & independent sampling)	Marine feeding area, return migration corr-idor, spawning & overwinter-ing area		Mid-late July; August; September (2008–2017)	Previously tagged	Live & dead^+^	95^d^

* specifically, whether migrating to the ocean or returning from an ocean migration

^#^ Two samples did not have reproductive status recorded when dead-sampled

^a^ Originally n = 129 however 17 may not have lived long enough to skip ocean migration for the first time and were therefore not used to investigate migration life history and its association with spawning frequency

^b^ 10 of these were part of the 17 omitted from migration life history analyses

^c^ Originally 60 but 11 of these were part of the 17 omitted from migration life history analyses and only 40 could be used to investigate relationship between skip and annual ocean migration life histories and spawning frequency

^d^ 82 female and 13 male.

^+^ The 40^c^ is part of the total 95.

It was possible to determine whether a fish would spawn in the current year or not when assessing maturity status (macroscopic visual evaluation of gonads) when dead-sampling in the summer and fall prior to spawning. A total of 129 recaptured fish were collected from the Shingle Point, Mackenzie Delta, and RR monitoring programs between 2008 and 2017, representing tagged fish that had been at-large between one and six years prior to recapture and presumably randomly mixed among the non-tagged component of the anadromous population. All samples with otoliths suitable for laser ablation (Sr concentration analysis), and with sex and maturity recorded in two different, sometimes successive, years (except for two without maturity and one without sex recorded upon recapture) were selected.

Tagged Dolly Varden were also recaptured in the annual seining program in Fish Creek during the fall (end of September) from 2008–2017 and live-sampled. All females (n = 82) and males (n = 13) tagged as spawners and recaptured in the following year in harvest monitoring (known to have migrated to sea in the current year) or seining programs (may or may not have migrated to sea in the current year) were used to determine the proportion that either rested or spawned to characterize the annual spawning frequency between sexes, although the migration status in the current year would remain unknown except for 40 samples used in otolith Sr analysis (Table 1).

### Collection of samples with unknown migration history in year of capture

Samples of unknown migratory history in the current-year were collected from the spawning/overwintering area and in the freshwater migration route (Fish Creek and Aklavik, Fig 1) not previously known to be occupied by anadromous individuals at the time of capture (Table 1) (S1 Table). Presumed anadromous Dolly Varden (i.e., either skipped migration or had already returned from an ocean migration) were collected from Fish Creek (spawning and overwintering area) on 18 July 2011 by angling in various pools in the same area where seining (tagging) occurred (note, water level and velocity were too high that day to effectively seine). A total of 27 fish were captured (FL range = 435–640 mm) of which four were recaptures from tagging in previous years. Nine were dead-sampled (two were recaptures) to confirm sex and reproductive status, record gonad weight (g), and collect otoliths for Sr analysis to confirm lifetime migration history; although only eight were suitable for laser ablation as otoliths in one sample were crystalline (i.e., annuli were illegible as the calcium carbonate of otoliths were deposited as vaterite rather than aragonite isoform [38]). Ovaries were frozen and later thawed and preserved in a 10% formalin solution for subsequent total egg count. Egg diameter was measured by taking the mean of three measurements of 10 randomly selected eggs aligned on a millimetre ruler. The gonadosomatic index (GSI; %) was calculated as: (gonad weight/total weight) x 100. A small sample size of fish was taken to reduce the impact on the stock due to past declines in abundance [39] and minimize interference with the voluntary subsistence harvest level for the stock.

Untagged anadromous Dolly Varden (i.e., travelling either to or from the sea) was captured by subsistence harvesters in Aklavik (Mackenzie Delta) on 24 June 2013 (n = 1) and 21June 2014 (n = 1) and reported to us independent from the harvest monitoring programs. Only the length data and head (otoliths) was provided in 2013 (FL = 610 mm) while the entire fish was supplied in 2014 (FL = 605 mm), which allowed for comprehensive biological sampling.

### Otolith ageing and laser ablation

One of each pair of otoliths was thin-sectioned (transverse plane through the nucleus) and aged according to the methods outlined in [40]. Annuli were identified based on the criteria described in [41] although weak and incomplete translucent bands were considered to be false annuli (“checks” [41]) and therefore not counted. All otoliths selected for laser ablation were re-embedded into acrylic rings, polished, cleaned, and photographed. Laser ablation inductively coupled plasma mass spectrometry (LA-ICP-MS) analysis (LUV 213 laser and Thermo Finnigan Element 2 ICP-MS) of otoliths was conducted at the Department Geological Sciences at the University of Manitoba. The ablation path was selected to perpendicularly cross annuli from the core region (primordium) to the outer edge of the dorsal lobe of the otolith to obtain annual ^86^Sr and ^43^Ca patterns throughout the entire life of the fish. The beam width was 30 μm and moved at a speed of 2 μm • s^-1^ with a repetition rate of 20 Hz. A NIST 610 glass standard was analyzed every hour, with 4 to 5 samples analyzed per hour, while Sr was internally standardized against calcium for ablation yield (constant Ca; in pure aragonite 40.02 wt%) and quantified against a NIST 610 external standard reference. Iolite (v. 2.21) [42] was used to convert Sr counts per second to parts per million (ppm) by correcting to the Ca and NIST 610 standards. A photograph was taken of each otolith following ablation.

### Migration interpretation and analyses

The Sr profiles were overlaid on photographs of ablated otoliths to visualize variation in concentrations among annuli. In the Fish Creek and Aklavik samples (i.e., unknown ocean migration history in year of capture), Sr concentration in the annulus deposited in the current summer was examined to confirm if a seaward migration was undertaken in the spring prior to dead-sampling (i.e., represented by a dramatic increase in values). Using Sr data from tagged fish recaptured in harvest monitoring programs, the Sr concentrations in the annuli corresponding to the year when a fish was tagged was determined by subtracting the years-at-large from the age of the fish to characterize the migration history and reproductive status for that specific age/year.

Evaluating parameters such as mean, minimum, maximum, and range of Sr among otolith annuli has been used to infer migration patterns between freshwater and marine habitats in anadromous salmonids [e.g., 43,44]. The following steps were conducted on all Dolly Varden examined for otolith Sr to differentiate between pre- and post-migratory periods, and between an ocean migration and possible skip migration event. For each age class (except age 0), the maximum and minimum Sr concentration in summer and winter growth zones, respectively, was determined. In age classes ≥1 years, the absolute difference between the maximum summer Sr concentration and the minimum value from the previous winter was calculated for each annuli (i.e., greatest difference in Sr concentration between summer and winter; hereafter referred to as Sr range). However, the oldest age class was excluded as the Sr concentration in the summer or fall would not be considered complete due to the lag time between absorption and deposition in otoliths that would result in an underestimate of the seasonal amplitude [45,46]. Anadromous northern salmonines undertaking their first migration to the sea typically demonstrate a dramatic increase in Sr in the summer growth zone of the annulus corresponding to age-at-first migration [47]. After confirming for each sample that the age with the highest ranking difference in Sr between summer and the previous winter corresponded to age-at-first migration, we excluded Sr data from pre-smolt annuli to only evaluate Sr in annuli ≥ age-at-first migration. We did not expect Sr levels in the annuli of post-smolt fish corresponding to winter months (i.e., narrow hyaline zone in an annulus [41]) when the fish would be occupying freshwater to return to pre-smolt concentrations as this has not been documented to occur in northern salmonid species that undertake annual migrations between freshwater and marine habitats [16,22,44,47–49].

Strontium range for each year following first migration was plotted against maximum Sr to identify annuli demonstrating low range in annual Sr relative to maximum Sr that would indicate a skip migration event. Plotting Sr range against maximum Sr has been used in other salmonids to identify unique migratory groups and elucidate variation in migration behaviour [e.g., 22,48]. A t-test was used to compare maximum Sr values in the current annulus of samples captured in mid-July from Fish Creek to the values from recaptured fish with low range in annual Sr as a means of determining whether samples from Fish Creek had skipped migration. A chi square test was used to evaluate if there was a significant relationship between spawning frequency and the migration life history when reproductive status of recaptured fish was known in two successive years. A Mann-Whitney U test was used to determine if age-at-first migration differed between migration life histories to test for ontogenetic effects. An unpaired t-test was used to determine if mean age differed between migration life histories while age frequency distributions were compared to infer differences in longevity. Statistical tests were conducted using R statistical computing package version 3.3.2 [50] and considered significant if p ≤ 0.05.

### Maternal habitat

Strontium concentration in the otolith core (primordium formed during embryonic development) of salmonids has been used to confirm whether the mother that produced the offspring occupied freshwater (low value) or marine (high value) habitat during vitellogenesis [51]. Strontium was compared between the primordia and first summer growth region to determine the maternal habitat occupied preceding spawning [52]. Using samples from harvest monitoring programs (n = 112) and Fish Creek (n = 8), apart from six that were excluded due to incomplete ablation of the core, an unpaired t-test was used to determine whether Sr was significantly higher in the core compared to the first summer growth region. Significant and non-significant differences would indicate the sample’s mother conducted or skipped, respectively an ocean migration in the year it was spawned. A chi square test was used to evaluate if there was a significant relationship between maternal pre-spawning habitat and the migration life history of the progeny.

## Results

### Samples with known migration history in year of recapture

**Fig 2:**
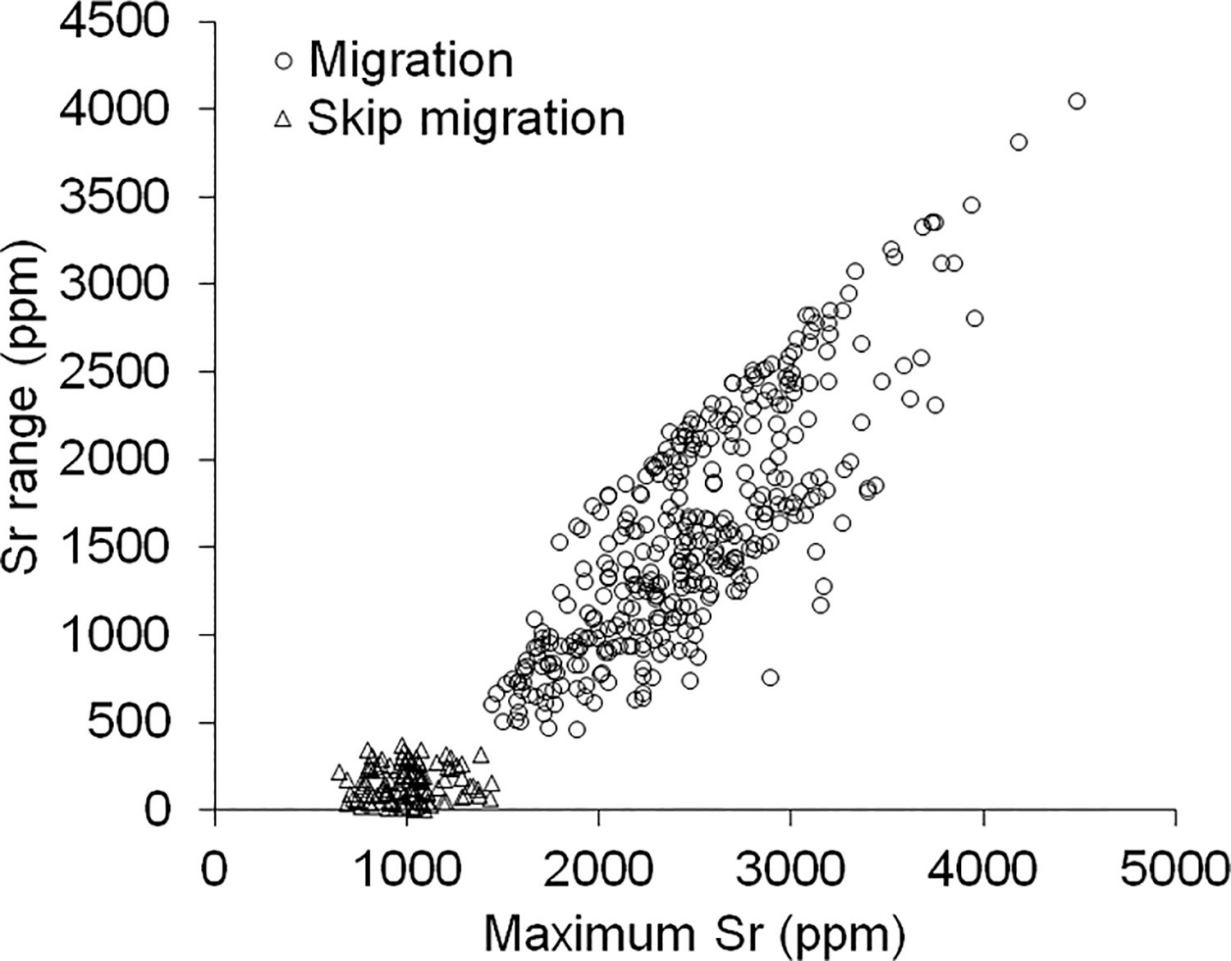
Strontium (Sr) range (difference in Sr concentration between the maximum summer and previous winter minimum values) plotted against maximum Sr among otolith annuli (only corresponding to years ≥ age-at-first migration) of anadromous Dolly Varden from the Rat River. Values are differentiated between instances when a skip ocean migration (open triangles; group 1) and ocean migration (open circles; group 2) event occurred.

Plotting the Sr range and maximum Sr among annuli ≥ age-at-first migration using the recaptures from harvest monitoring programs (n = 129) revealed two distinct groups of Sr concentrations among annuli ≥ age-at-first migration (group 1- low Sr range and low maximum Sr: range = 0.4–367 ppm, 647–1439 ppm, respectively; group 2- high Sr range and high maximum Sr: range = 458–4047 ppm, 1447–4488 ppm, respectively) (Fig 2). Strontium concentrations delineating group 1 and group 2 were considered to reveal incidences where skip migration and seaward migration occurred, respectively in an annulus.

**Fig 3:**
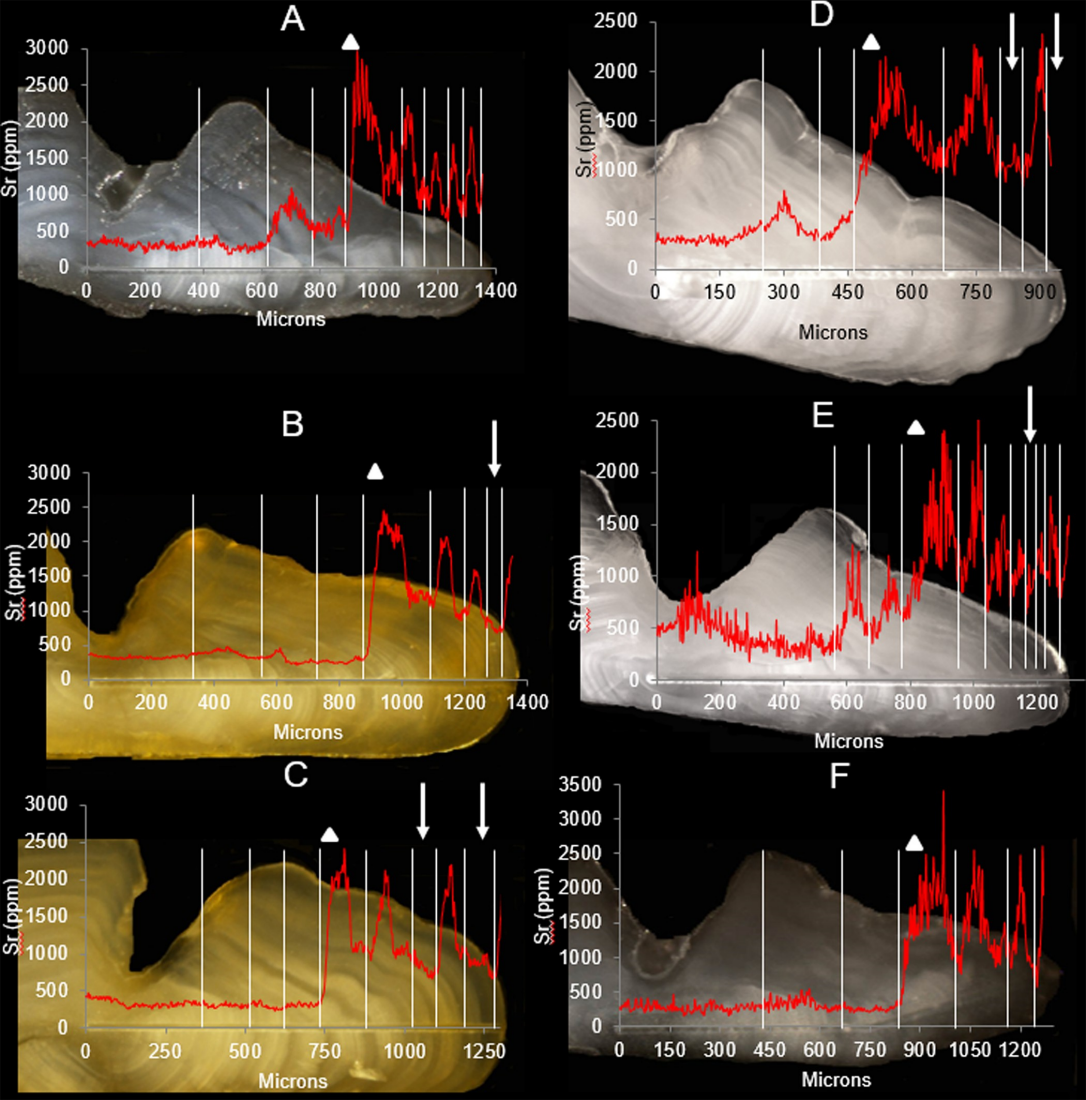
Strontium (Sr) (red line) overlain on images of ablated otoliths of anadromous Dolly Varden from the Rat River collected in: harvest monitoring programs during the summer and known to have undertaken an ocean migration in year of capture and have either (A) conducted annual migrations upon smoltification, or skipped ocean migration (B) once or (C) twice in the past; Fish Creek in mid-July that (D) skipped ocean migration or (E) returned early from ocean migration in the current year; (F) Aklavik in June that was returning early from an ocean migration. White vertical bars illustrate annuli locations; white triangles show smoltification events; white arrows show instances of skip migration. Note, Sr concentrations as high as 1814 ppm have been observed in the resident life history of this stock, therefore elevated values prior to smoltification are presumably not indicative of migration and estuarine habitat use.

**Fig 4:**
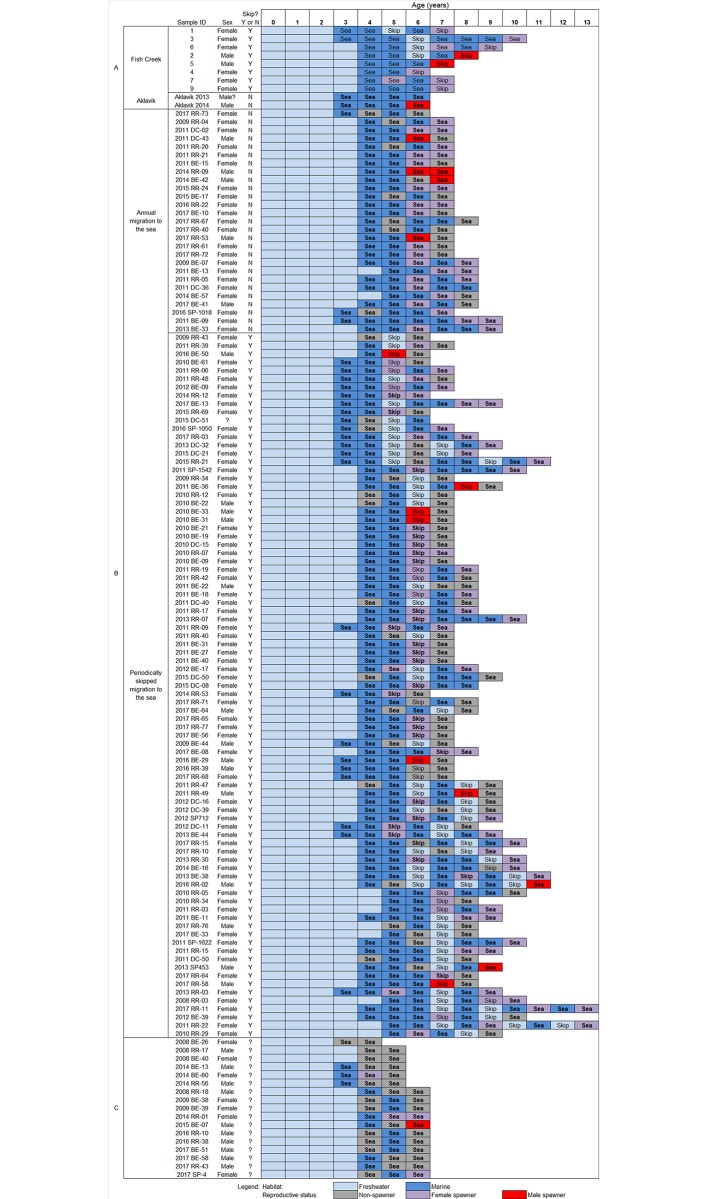
Reconstruction of habitats occupied annually over lifetime, noting incidence of migrations to the sea (‘sea’) and skip migration (‘skip’), and years when reproductive status (non-spawner, female spawner, male spawner) was known, in Dolly Varden from the Rat River. . Samples are sorted by those (A) with unknown migration history at time of capture (further sub-divided by capture location), (B) known to have migrated in the current-year at time of capture (further sub-divided by fish that either migrated to sea annually or skipped migration at least once during their lifetime; sorted by age at first skip migration), and (C) undetermined migration life history (i.e., may not have lived long enough to skip migration for the first time). Sample ID in B and C = year of dead-sample, site, fish number [site: SP = Shingle Point, BE = Big Eddy (Husky Channel in Mackenzie Delta), RR = mouth of Rat River, DC = Destruction City (Rat River)].

Strontium profiles from tagged fish recaptured in harvest monitoring programs revealed two migration tactics with fish that either migrated annually (Fig 3A) or periodically skipped annual migration (Fig 3B and 3C) in some years after smoltification (Fig 4). Annually migrating fish accounted for a smaller proportion (24.1% of n = 112) of the sample compared to those who skipped migration in some years (75.9% of n = 112). Given that 19.4% (of n = 93) of first instances of skip migration occurred after completing three or four migrations, 17 of the 129 samples were omitted from estimating the proportion of either migratory life history as these had only gone out to sea three consecutive times upon smolting and may not have lived long enough to skip for the first time (resulting in n = 112 samples). No significant difference in age-at-first migration was detected between fish that either did or did not conduct skip migration (U = 1136, p = 0.34).

### Samples with unknown migration history

Dolly Varden captured in Fish Creek in mid-July ranged between ages six and 10 years and were starting to adopt spawning colouration while the gonads (seven females and two males) confirmed they would spawn in the current year (female: mean ± 95% C.I. egg diameter = 3.3 ± 0.2 mm, GSI = 8.9 ± 2.9%; male: GSI = ~5%) (i.e., egg diameter and GSIs were within range of current-year spawners [14]). Strontium concentrations among the eight samples confirmed migrations to the sea in previous years, however concentrations at the otolith edge (deposition during the summer of capture) indicated seven had remained in freshwater over the summer (Fig 3D) while one (female) had already returned to the spawning grounds after undertaking a migration to the sea that summer (Fig 3E). The Sr concentration in the annuli of the seven Fish Creek samples under deposition at the time of dead-sampling were not significantly different from Sr values in the low Sr range and low maximum Sr group associated with skip migration events (Fig 2) (t = 0.65, d.f. = 118, p = 0.52).

Both Dolly Varden captured in Aklavik in June were six years of age, smoltified at age three, and conducted consecutive-year migrations to the sea thereafter. Strontium concentrations at the section edge in the 2013 sample revealed no increase relative to the winter growth while a dramatic increase was evident in the 2014 sample (Fig 3F). The 2013 sample had remnants of a kype suggesting it was a male while the 2014 sample was a male current-year spawner with a GSI of 7.5%.

### Skip migration behaviour

**Fig 5:**
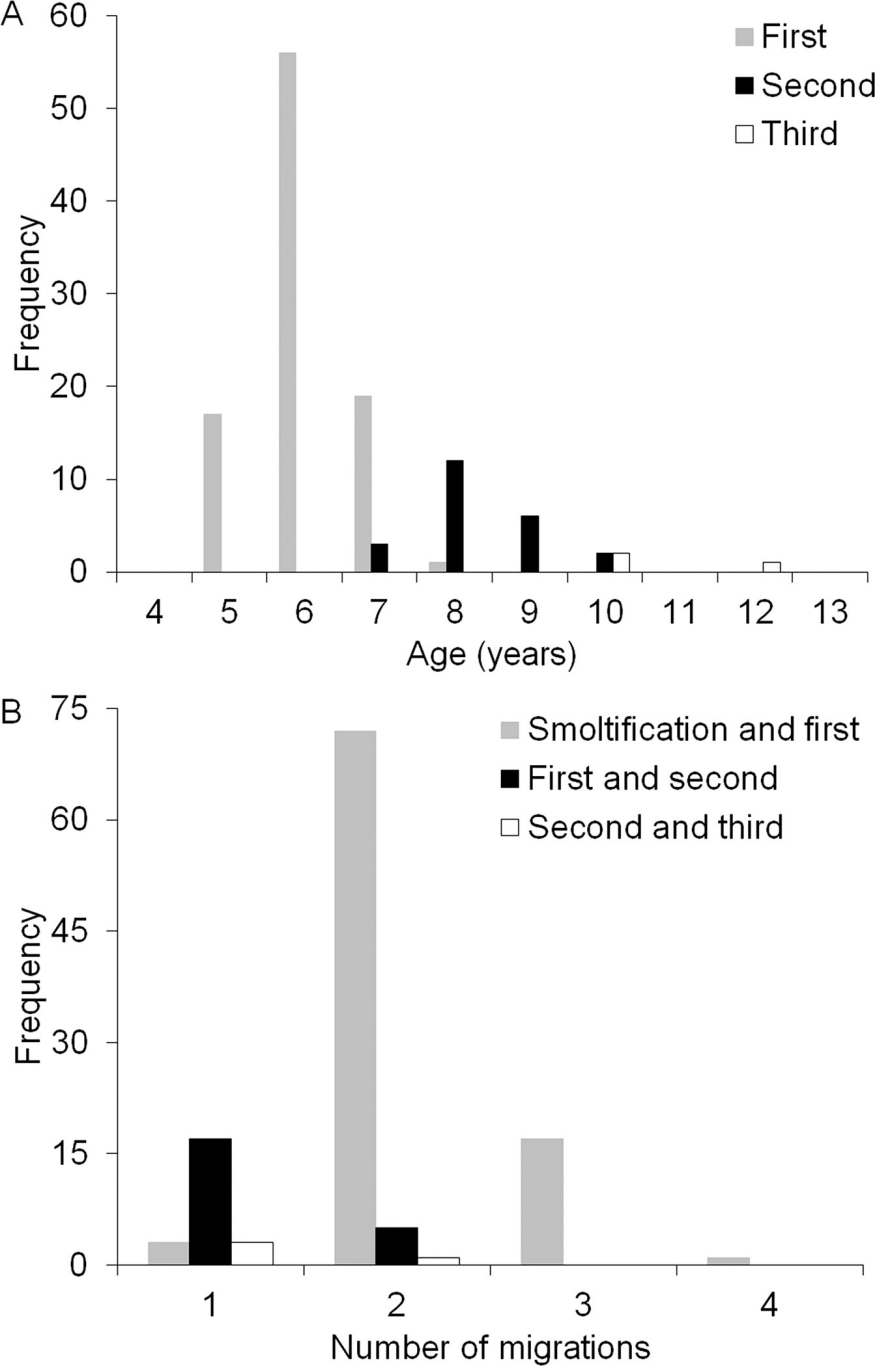
(A) Frequency of age of first, second and third occurrence of skip ocean migration, and (B) number of seaward migrations between smoltification and first skip migration, first and second skip migration, and second and third skip migration events in anadromous Dolly Varden from the Rat River.

Among those who skipped migration during their lifetime (n = 93; Fish Creek samples n = 8 and subsistence fishery recaptures n = 85), the majority (74.2%) had performed this only once (Fig 3B and 3E), however approximately one fifth (22.6%) skipped twice (Fig 3C and 3D), while only a small proportion (3.2%) skipped three times. All incidences of skipped migration were followed by a migration to the sea in the subsequent year. Most Dolly Varden skipped migration for the first time at age six (60%), although this was observed in fish as young as five and as old as eight years of age (Fig 5A). Ages of the second skipped migration was typically eight years (52.2%) while the third skipped migration only occurred among older age classes (≥10 years) (Fig 5A). The number of seaward migrations undertaken between smoltification and the first skip ranged between one and four with most (77.4%) occurring upon completing two migrations. Likewise, the number of seaward migrations between the second and third skip migration event was between one and two (Fig 5B).

### Migratory life history, spawning frequency, and longevity

Based on all of the examined samples (n = 139), age-at-first migration ranged between three (21.6%) and five (7.2%) years with the large majority (71.2%) of occurrences at age four. Using all available samples, there were 131 instances where it was possible to match the reproductive status to the same year as a skip migration (regardless of whether it was the first, second or third time) and or the year preceding and following the skip, while simultaneously evaluating the migration history in the year before and after the skip. Most Dolly Varden (90.7%) were in spawning condition during the year they skipped migration while 9.3% were ‘non-spawners’ (Table 2). In the year prior to and after skip migration, a large majority were identified as ‘non-spawner’ (85.7% and 69.8%, respectively) (Table 2).

**Table 2:** Frequency (% in brackets) of anadromous Dolly Varden from the Rat River exhibiting skip ocean migration life history that were identified as a ‘spawner’ or ‘non-spawner’ before, during, and after the skip migration event.

Reproductive status	Before	During	After
Non-spawner	12 (85.7)*	5 (9.3)	44 (69.8)
Spawner	2 (14.3)^+^	49 (90.7)	19 (30.2)
Total	14 (100)	54 (100)	63 (100)

*10 instances prior to the first skip and presumed to be juveniles, and two instances between first and second skip migration events and presumed to be adults

^+^one instance prior to first skip and the other prior to second skip.

Instances when reproductive status was determined in two successive years (n = 40) in both the skip (n = 26) (i.e., when the reproductive status was known during the skip migration and subsequent ocean migration the following year) and annual (n = 14) migration life histories demonstrated significantly different spawning frequencies between the two types (χ^2^ = 5.47, p = 0.019). Although repeat and alternate-year spawning were observed in both migratory life histories, the incidences of alternate-year spawning was higher in fish that skipped ocean migration (84.6%) compared to fish that migrated to the ocean annually (50%) (Table 3). All annual migrants were identified as ‘spawner’ in at least one year during their lifetime after smoltification.

**Table 3:** Frequency (% in brackets) of repeat and alternate-year spawning in anadromous Dolly Varden from the Rat River tagged as current-year ‘spawner’ and recaptured the following year as either a current-year ‘spawner’ (i.e., repeat spawning) or ‘non-spawner’ (i.e., alternate-year spawning) in fish that either exhibited skip ocean migration (i.e., skipped migration and conducted an ocean migration the following year in the same two-year span when reproductive status was known) or annual ocean migration life history.

Migration life history	Repeat spawning	Alternate-year spawning	Total
Skip migration	4 (15.4)	22 (84.6)	26 (100)
Annual migration	7 (50)	7 (50)	14 (100)

In some instances, Dolly Varden demonstrating skip migration also performed consecutive annual migrations to the sea after the skip event (n = 36). Most completed two consecutive migrations (75%) while fewer (25%) conducted three or four annual migrations in a row before skipping again. Among individuals where reproductive status was known in one of the years of consecutive migrations (n = 24), most were identified as ‘spawner’ (87.5%) while repeat ‘spawners’ (n = 3 females), repeat ‘non-spawner’ (n = 1 male), and alternate-year ‘spawners’ (n = 2 female) were detected when reproductive status was known in consecutive years.

Eighty-two females and 13 males tagged as current-year ‘spawners’ were recaptured between 2008 and 2017 after being at large for approximately one year. Spawning frequencies differed significantly between sexes (χ^2^ = 5.07, p = 0.02) with an equal proportion of females performing alternate-year (51.2%) and repeat (48.8%) spawning and a higher proportion of males performing alternate-year spawning (84.6%).

**Fig 6:**
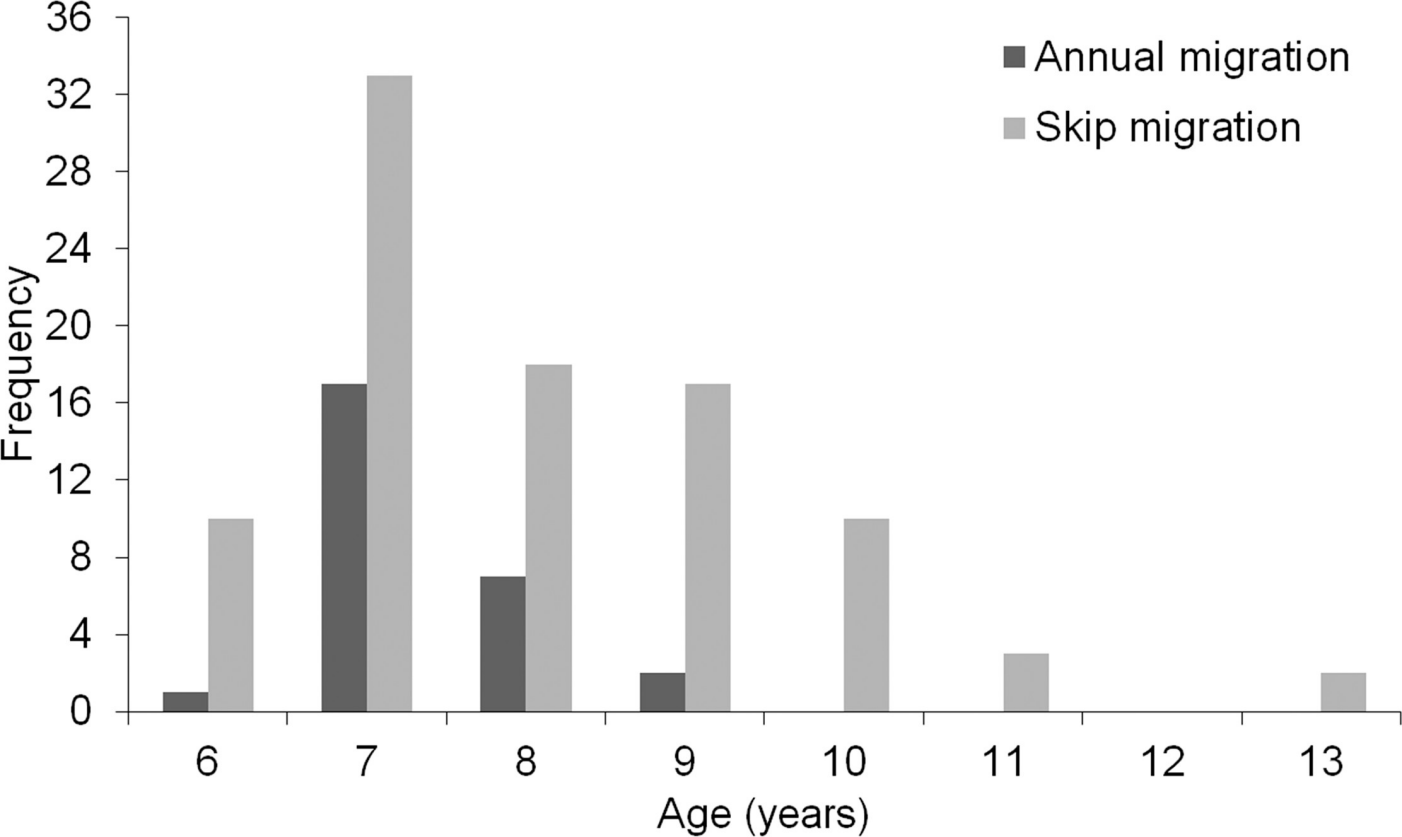
Age distribution of anadromous Dolly Varden from the Rat River that exhibited either a skip or annual ocean migration life history.

Age structure was significantly different between Dolly Varden that skipped annual ocean migration and those who did not (t = -2.21, d.f. = 118, p = 0.03). Fish that skipped migration had a higher mean age (8 years), a higher proportion among older age classes, and reached a greater maximum age (13 years) (Fig 5). Alternatively, annually migrating fish had a lower mean age (7.4 years) and a lower maximum age (9 years) (Fig 6).

### Maternal habitat

Strontium concentrations in the core were significantly higher (all p< 0.0001) compared to the first summer growth region in 80 of the 114 samples indicating the mothers of these fish undertook seaward migration prior to spawning. Mean ± SD of the core and first summer growth region were 637 ± 201 and 351 ± 78 ppm, respectively indicating the mothers undertook seaward migration prior to spawning, and 377 ± 99 and 334 ± 84 ppm, respectively indicating mothers remained in freshwater prior to spawning. The pre-spawning maternal migratory status of the progeny that exhibited skip or annual migration tactics revealed no significant association between migratory life history and maternal pre-spawning habitat (χ^2^ = 0.26, p = 0.61) (Table 4).

**Table 4:** Frequency (% in brackets) of strontium concentrations in otolith primordium indicating whether a Dolly Varden from the Rat River exhibiting either skip or annual migration tactics was spawned from a mother that had migrated (marine) or not (freshwater) prior to spawning*.

Migration life history	Maternal habitat preceding spawning		Total
	Marine	Freshwater	
Skip migration	60 (69)	27 (31)	87 (100)
Annual migration	20 (74.1)	7 (25.9)	27 (100)

* n = 17 fish with three consecutive migrations upon smolting were omitted as these may have been dead-sampled prior to first skip.

## Discussion

The combined use of mark-recapture and otolith Sr to associate multi-year reproductive condition with lifetime migration histories in this study provided a novel approach for revealing two migratory life histories and a complex relationship between migration and reproduction not previously observed within a salmonid population. The two migratory life histories were 1) skip migration, where a fish will periodically forgo seaward migration in spring and remain in freshwater to spawn but resume migration the following spring, and 2) annual migration, where a fish conducts consecutive annual migrations to the sea upon smoltification. Our study revealed four categories of association between migration and spawning frequency: 1) spawn during skip migration and rest the following year after ocean migration, 2) spawn during skip migration and spawn the following year after ocean migration, 3) migrate annually and spawn in consecutive years (at least two), and 4) migrate annually and spawn in alternate-years (at least two). The latter two were observed in both annual and skip (i.e., during consecutive-year migrations after a skipped migration event) migration life history types. Skip migration appeared to be highly associated, although not exclusively, with alternate-year spawning behaviour while annual ocean migration was associated equally with repeat and alternate-year spawning behaviour. Our results indicate that Fish Creek is the freshwater location where fish remain when Dolly Varden from the RR population forgo ocean migration. Further complexity in migration behaviour was exhibited by fish that returned from the sea considerably earlier than the majority of migratory component of the population (i.e., a 2011 Fish Creek sample (Fig 3E) and the 2013 sample from Aklavik (Fig 3F) had and was, respectively returned/returning considerably early from an ocean migration), an event that is poorly documented in Dolly Varden.

The common pattern for skip migration in Dolly Varden from the RR is as follows: upon smolting at age four and conducting migrations two years in a row, a fish will skip migration and remain in spawning/overwintering habitat (i.e., Fish Creek), which is consistent with systems in Alaska [18–20], and presumably spawn for the first time (age 6 years). Subsequently, a seaward migration is required the following year to replenish the energy spent on the previous return migration and maintenance during two overwintering periods with a spawning event in between (i.e., ~22 months without marine feeding). Upon the return migration (upstream direction) after the first skip, most Dolly Varden did not spawn (i.e., they were resting) (Table 2) although in some instances individuals appeared to accumulate enough energy to spawn in the current year (i.e., spawn in consecutive years). Subsequently, fish either skipped migration again (typically at age eight) and spawned or continued to conduct annual seaward migrations (predominantly two consecutive migrations) with the potential to spawn, or not, after completing these migrations until skipping again or death. Exceptions were two fishes that spawned in the year prior to skip migration (sample ID: 2011 RR-022, 2012 BE-017; Fig 4) and five that did not spawn in the year they skipped migration. It seems unlikely the two fish would have spawned during the skipped migration given the high energetic demands of spawning and overwintering twice without going to sea. A possible explanation was incorrect ageing and assignment of ages with years when reproductive status was known (it is noted that recaptured samples were at large for 3–4 years). It is unclear why the five fish did not spawn in the year they skipped migration, although it may be due to surpassing an energetic threshold that triggered a ‘decision’ to skip migration yet a threshold (e.g., nutritional or environmental) to initiate spawning was not met [12].

Further research (e.g., genetic analyses) is required to conclusively determine whether both migration tactics are facultative or obligatory. However, several lines of evidence suggest they are facultative, specifically: no difference in age-at-first migration, lack of association between life history and maternal pre-spawning habitat, and the ability of individuals who skip an ocean migration to spawn either during the skip migration event or in the same year as a migration. Facultative anadromy has been observed in other populations of Dolly Varden [e.g., 23], however confirming whether the two anadromous migration tactics of the RR population are obligatory would be beneficial to inform fisheries management objectives to protect and conserve life history variants that are vulnerable to subsistence fisheries (see [53]).

As predicted, different migration tactics produced trade-offs in spawning frequency and longevity. Specifically, those who skipped migration were more likely to spawn in alternate years and live longer than those who migrated annually. While annually migrating fish had lower longevity, trade-offs included increased possibility of annual reproduction (relative to fish who do not migrate annually) and opportunity for growth. Although our sample size is small for annually migrating Dolly Varden that had reproductive status known two years in a row, the equal representation of repeat and alternate-year spawning in these fish suggest the energetic and mortality costs of annual migration have less influence on reproductive frequency compared to fish that skipped migration. Alternatively, those who skipped migration may experience reduced probability of mortality during each skipping event while simultaneously reproducing, as hypothesized by [20], which may promote greater longevity; although little to no feeding for ~22 months and spawning between overwintering years could also result in high mortality [54]. However, trade-offs likely include limited growth during the skip migration with the associated need to migrate in the following year and invest in growth rather than reproduction, although a minority gain enough energy during this migration to spawn two years in a row (Table 3).

If migration life history is facultative, it is unclear why most of the skip migration events occurred at age six and mainly happened once during the lifespan of the majority of the fish examined. The frequency and pattern of alternate-year spawning should result in a trade-off between current and future reproductive success influenced by mortality, food intake, and migration costs [9]. We speculate that the pattern of alternate-year spawning and skip migration observed in Dolly Varden from the RR provides the greatest opportunity for maximizing fitness (i.e., life time reproductive output). Age six is equal to age at maturity in both sexes and a point in the life history where 47% and 59% annual mortality can be expected by females and males, respectively in the population (S1 Fig). The mortality associated with the onset of maturity may be reduced by skipping a feeding migration to maximize the likelihood of survival and reproductive output in a relatively large component of the adult migratory population while the frequency of additional skipping events would depend on the longevity of skip migrants, which is relatively low given the rapid decline in the proportion of fish ≥8 years (S1 Fig). Furthermore, research is needed to elucidate whether inter-annual variation in the marine and freshwater environment influences skip migration patterns in Dolly Varden to determine whether skip migration events are a response to conditions (e.g., marine prey availability and quality, water temperature) that influence energetic accumulation in fish and its use for investment in migration and reproduction. Determining if there is an association between the prevalence of a migration tactic in a given year with condition factor (K), an indicator of energetic accumulation, may help reveal climate-related influences given that higher condition factor in Dolly Varden from the RR is positively correlated with earlier dates of ice breakup along the Beaufort Sea coast [36].

The trade-offs between migration and reproduction in both migratory life histories would presumably differ between females and males given that migratory tendency is predicted to differ between sexes when the cost and benefits of migration are unequal [8]. Although further study is required to statistically evaluate whether female and male Dolly Varden exhibit differences in frequency of skip migration, the higher prevalence of alternate-year spawning in males found in our study suggests a high cost associated with the investment in secondary sexual characters (e.g., development of kype, dorsal hump, spawning colouration, enlarged teeth, and lateral compression) and defending potential mates and spawning territory. The high cost of reproduction would require a trade-off against other energetic expenses [55] such as repeat-spawning (although see [56]). Alternate-year spawning is hypothesized to be advantageous in fishes with energetically costly reproductive behaviour [57]. Given the presumably high investment in reproduction and demonstrated higher annual mortality (female = 47%, male = 59%) (S1 Fig), it is plausible that males may skip migration more often than females as a means to decrease the likelihood of mortality during migration to protect their investment. Although some males spawn in the same year they migrate (all but one occurrences was during consecutive-year migrations), resting in the year after skipping migration would contribute to growth that is beneficial for defending optimal spawning habitat. In females, the equal incidence of skip and annual spawning combined with the high (75.9%) frequency of skip migration that typically results in alternate-year spawning suggests females have higher incidences of annual spawning during consecutive-year migrations to the sea (regardless of migration life history) compared to males.

In western Alaskan systems where Dolly Varden skipped migration to spawn, separate spawning populations (‘summer’ and ‘fall’ spawners) have been described [18,19]. ‘Summer spawners’ skipped migration and remained in freshwater and spawned earlier compared to ‘fall spawners’ that went to sea and returned in the fall to spawn [18]. While our study is the first to confirm skip migration in Canadian Dolly Varden stocks, further research is required to determine if fish from the RR that skip migration and the migrants that return considerably earlier also spawn earlier than those returning from the sea during the ‘normal’ migration period or whether both groups of fish reproduce in a single temporally protracted spawning period in Fish Creek (~early/mid-August to ~mid-September).

The two Dolly Varden captured in Aklavik in late June presumably belonged to the RR population [27]. While one fish was confirmed to be returning from a migration at sea, the migration status of the other was ambiguous. The fish was either migrating towards the sea or was returning from the sea yet the time lag between the absorption and deposition of Sr in otoliths [46] compounded by a potential high rate of swimming (e.g., moving 51 km in five days in the Mackenzie Delta; [58]) and short marine feeding duration prevented confirmation of marine habitat use. 21 June 2014 is the earliest documented date of capture in the Mackenzie Delta of a Dolly Varden confirmed to be returning from the sea. This sample combined with the one sample that returned to Fish Creek by mid-July indicates some individuals from RR population undertake the long freshwater migration to spend a presumably short amount of time in the ocean and return in a pre-spawning state to spawning grounds considerably earlier relative to other migrants. This “premature migration” strategy is poorly documented in Dolly Varden but is hypothesized to occur so individuals can gain access to seasonally constrained breeding sites and or may arise from a trade-off between opportunity for growth against probability of mortality [59]. Prior to 2013, no Dolly Varden had been harvested in late June in Aklavik (William Storr, Aklavik, personal communication). Harvesters consider upstream migration as “early” if fish are captured in the second week of July which involve a few moving upstream over the course of a couple of weeks until greater numbers are captured in late July (William Storr, personal communication).

This study suggested that alternate migration tactics affected spawning frequency and longevity in anadromous Dolly Varden, an iteroparous salmonid that can undertake long distance migrations through complex habitats (i.e., small streams and large delta systems). The association between skip migration and alternate-year spawning and the higher prevalence of alternate-year spawning in males suggest fitness trade-offs between migration life histories differed between the sexes. Combined with the alternative reproductive tactics (i.e., stream resident life history), the diversity of reproductive and migratory life histories, and timing in return migration observed in the anadromous component underscores a bet hedging strategy used by the RR population to promote resilience. Indeed, additional work is necessary to evaluate the effects of distance on migration patterns and spawning frequency using analogous data in Dolly Varden inhabiting shorter river systems and to evaluate the trade-offs in migration (e.g., growth, condition, and fecundity) to determine mechanisms of skip and annual migration, and alternate-year and repeat spawning. This study provides a contribution towards understanding intrapopulation diversity in migration behaviour and reproductive frequency, traits that have implications for population productivity; a pertinent issue given northern Dolly Varden has been listed as ‘Special Concern’ under the *Species at Risk Act* in Canada.

## Supporting information

S1 Table Sample characteristics of the mark-recapture study conducted between 2007 and 2017 to examine migration tactic and spawning frequency of anadromous Dolly Varden from the Rat River. (PDF)

S1 Fig Age frequency of female (n = 971) and male (n = 715) anadromous Dolly Varden from the Rat River captured using gill nets and sampled by the Rat River Harvest monitoring program between 2008 and 2017. Annual mortality (A; ± 95% confidence intervals) for both sexes calculated using Robson-Chapman method [60].
(PDF)

S2 Fig Male (top) and female (second from the top) anadromous Dolly Varden in spawning condition. Note, two pre-smolt juvenile Dolly Varden below. Photo by C. Gallagher.
(DOCX)
