# Supplementary material for: Migration tactics affect spawning frequency in an iteroparous salmonid (Salvelinus malma) from the Arctic
Source: PLoS One. 2018 Dec 31;13(12):e0210202. doi: 10.1371/journal.pone.0210202 (PMC6312342; doi:10.1371/journal.pone.0210202)
Supplement: S1 Table — (PDF) [file pone.0210202.s001.pdf]

S1 Table. Sample characteristics of the mark-recapture study conducted between 2007 and 2017 to examine migration tactic and spawning frequency of anadromous Dolly Varden from the Rat River. (A) Number of Dolly Varden captured with a seine net in Fish Creek (spawning and overwintering habitat and tributary to the Rat River; see open white circle in Fig 1) in fall and of these the number of recaptures of tags deployed in previous years and number of fish that were tagged (t-bar tags) (no tags deployed in 2008, 2011, and 2012) (all captured fish were released). (B) Number of Dolly Varden with 'known' and 'unknown' ocean migration history in year of capture encountered: in the Rat River (late summer to early fall; see closed circle 2, 3, and 4 in Fig 1) and Shingle Point (summer; see closed circle 1 in Fig 1) harvest monitoring programs, while angling in Fish Creek (mid-July 2011), and in subsistence fisheries in Aklavik (late June 2013 and 2014) (Fig 1). For 'known' samples, the reported number of fish captured in gill nets and of these the number of recaptures of tags deployed in previous years with the number of fish with otoliths, sex, and reproductive status data that were used in our study in [square brackets]. For 'unknown' samples, the number of fish encountered where the number of fish with otoliths, sex, and reproductive status data used in our study in [square brackets] (no sex and reproduction data for Aklavik sample collected in 2013). Note, no formal harvest monitoring program at Shingle Point prior to 2011.

	A			B					
				Known ocean migration in year of recapture				Unknown ocean migration in year of capture	
	Fish Creek			Rat River		Shingle Point		Fish Creek	Aklavik
	Captured	Recaptures	Tagged	Captured[1]	Recaptures	Captured	Recaptures		
2007	432	6	426	-	-	-	-		
2008	522	13	0	461	10 [5]	-	-		
2009	470	8	462	250	13 [7]	-	-		
2010	471	39	431	357	16 [13]	-	-		
2011	157	19	0	482	35 [31]	193	2 [2]	27 [9*]	
2012	394	26	0	407	6 [6]	412	1 [1]		
2013	463	7	456	302	9 [7]	115	1 [1]		1 [1]
2014	539	20	499	441	12 [10]	404	3 [0]		1 [1]
2015	542	31	496	762	28 [9]	375	6 [0]		
2016	545	37	495	490	9 [7]	313	7 [2]		
2017	-	-	-	607	35 [27]	181	4 [1]		
Sum[+]					[122[a]]		[7[b]]	[8]	[2]
Total[#]					[106]		[6]	[8]	[2]

[1] some fish were released in an attempt not to surpass annual voluntary harvest levels established for the population

* 8 samples were suitable for otolith strontium analysis

[+] examined for otolith strontium concentration

[a]16 and [b]1 samples were omitted from analyses as these may not have lived long to skip ocean migration for the first time

[#] total number of samples used to evaluate migration tactic= 122
