# Supplementary material for: Migration tactics affect spawning frequency in an iteroparous salmonid (Salvelinus malma) from the Arctic
Source: PLoS One. 2018 Dec 31;13(12):e0210202. doi: 10.1371/journal.pone.0210202 (PMC6312342; doi:10.1371/journal.pone.0210202)
Supplement: S1 Fig — Annual mortality (A; ± 95% confidence intervals) for both sexes calculated using Robson-Chapman method [60]. (PDF) [file pone.0210202.s002.pdf]

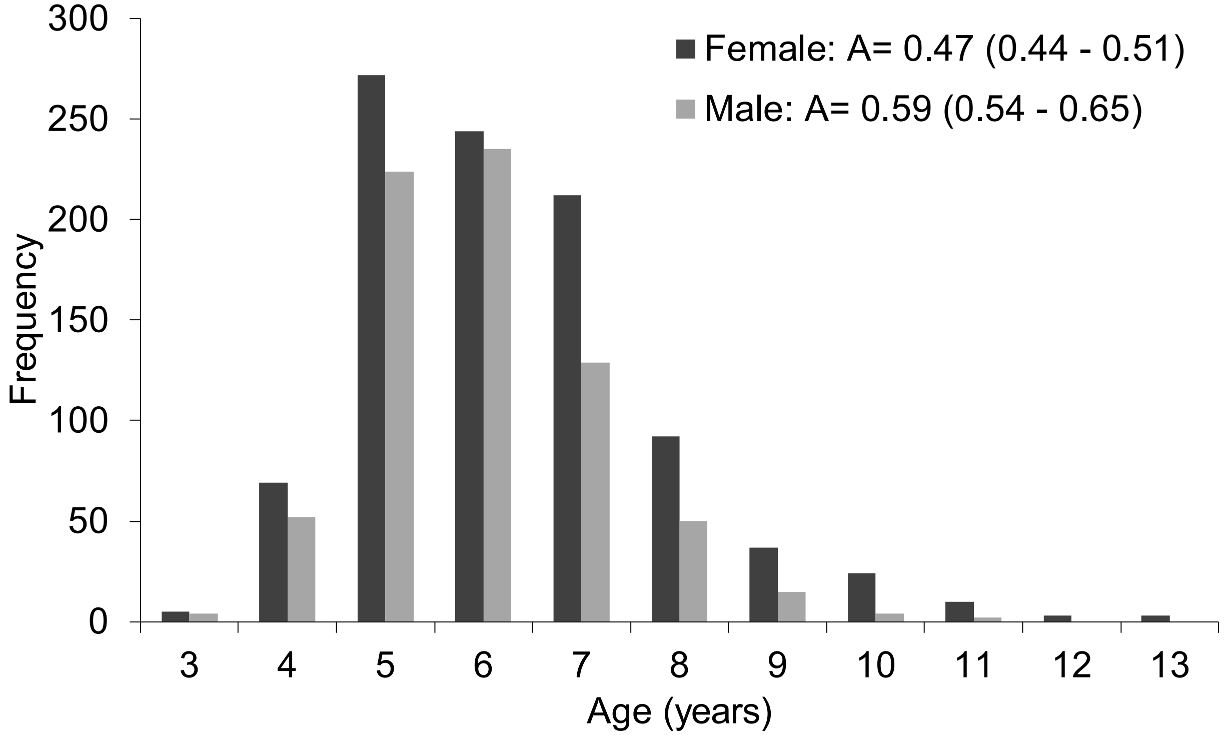

S1 Fig. Age frequency of female (n= 971) and male (n= 715) anadromous Dolly Varden from the Rat River captured using gill nets and sampled by the Rat River Harvest Monitoring Program between 2008 and 2017. Annual mortality (A; ± 95% confidence intervals) for both sexes calculated using Robson-Chapman method [60].
