# Supplementary material for: Migration tactics affect spawning frequency in an iteroparous salmonid (Salvelinus malma) from the Arctic
Source: PLoS One. 2018 Dec 31;13(12):e0210202. doi: 10.1371/journal.pone.0210202 (PMC6312342; doi:10.1371/journal.pone.0210202)
Supplement: S2 Fig — Male (top) and female (second from the top) anadromous Dolly Varden in spawning condition. Note, two pre-smolt juvenile Dolly Varden below. Photo by C. Gallagher. (DOCX) [file pone.0210202.s003.docx]

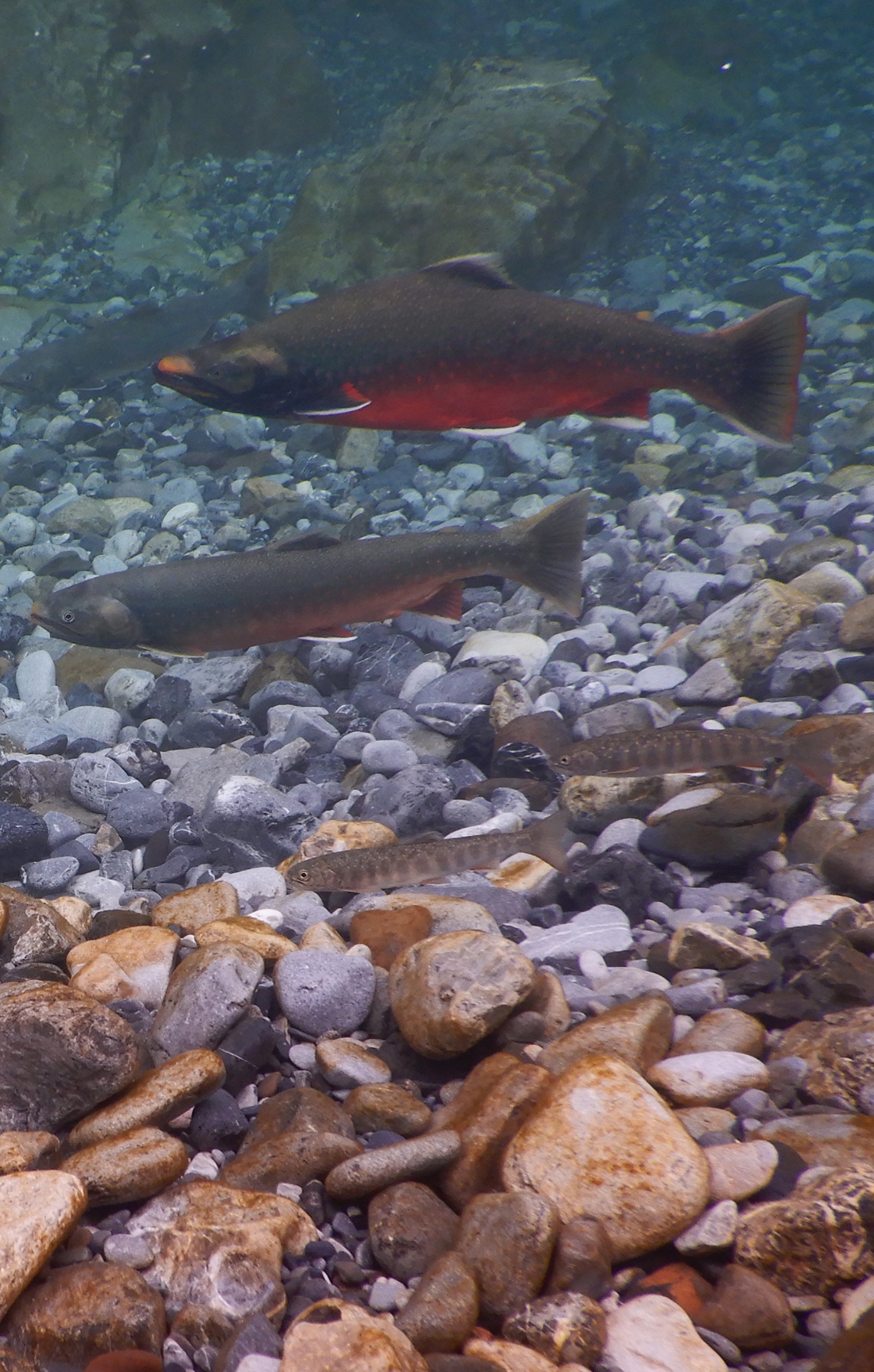


S2 Fig. Male (top) and female (second from the top) anadromous Dolly Varden in spawning condition. Note, two pre-smolt juvenile Dolly Varden below. Photo by C. Gallagher.
